# The effects of signalment, diet, geographic location, season, and colitis associated with antimicrobial use or *Salmonella* infection on the fecal microbiome of horses

**DOI:** 10.1111/jvim.16206

**Published:** 2021-07-16

**Authors:** Carolyn E. Arnold, Rachel Pilla, M. Keith Chaffin, Jessica L. Leatherwood, Tryon A. Wickersham, Todd R. Callaway, Sara D. Lawhon, Jonathan A. Lidbury, Joerg M. Steiner, Jan S. Suchodolski

**Affiliations:** ^1^ Department of Large Animal Clinical Sciences College of Veterinary Medicine & Biomedical Sciences, Texas A&M University College Station Texas USA; ^2^ Department of Small Animal Clinical Sciences College of Veterinary Medicine & Biomedical Sciences, Texas A&M University College Station Texas USA; ^3^ Department of Animal Science Texas A&M University College Station Texas USA; ^4^ Department of Animal and Dairy Science University of Georgia Athens Georgia USA; ^5^ Department of Veterinary Pathobiology College of Veterinary Medicine & Biomedical Sciences, Texas A&M University College Station Texas USA

**Keywords:** antibiotic, diarrhea, gastrointestinal, microbiota

## Abstract

**Background:**

The fecal microbiome of healthy horses may be influenced by signalment, diet, environmental factors, and disease.

**Objectives:**

To assess the effects of age, breed, sex, geographic location, season, diet, and colitis caused by antibiotic use (antimicrobial‐associated diarrhea [AAD]) and *Salmonella* infection on fecal microbiota.

**Animals:**

Healthy horses (*n* = 80) were sampled from nonhospital environments across multiple geographical locations in the United States. Horses with AAD (*n* = 14) were defined as those that developed diarrhea secondary to antimicrobial use. Horses with *Salmonella* infection (*n* = 12) were presented with spontaneous onset of colitis and subsequently tested positive on *Salmonella* quantitative polymerase chain reaction. All horses were >1 year of age and stratified by a dietary scale that included forages (pasture and hay) and concentrates grouped by percentage of fiber and amount.

**Methods:**

Illumina sequencing of 16S rRNA genes was performed on fecal DNA.

**Results:**

Healthy horses fed higher amounts of grain clustered separately from those fed lower amounts of grain (analysis of similarities [ANOSIM], *R* = 0.356‐0.385, *Q* = 0.002). Horses with AAD and *Salmonella* had decreased richness and evenness compared to healthy horses (*P* < .05). Univariable analysis of the 3 groups identified increases in Bacteroidetes (*Q* = 0.002) and Protebacteria (*Q* = 0.001) and decreases in Verrucomicrobia (*Q* = 0.001) in AAD horses whereas *Salmonella* horses had less Firmicutes (*Q* = 0.001) when compared to healthy horses.

**Conclusions and Clinical Importance:**

Although the amount of grain in the diet had some impact on the fecal microbiome, colitis had a significantly larger influence. Horses with ADD have a more severe dysbiosis than do horses with *Salmonella*.

**Abbreviations**
AAD: antimicrobial associated diarrhea
ASV: amplicon sequence variant
ANOSIM: analysis of similarities
DADA2: divisive amplicon denoising algorithm
DNA: deoxyribonucleic acid
NSAID: nonsteroidal anti‐inflammatory
qPCR: quantitative polymerase chain reaction
QIIME: Quantitative Insights into Microbial Ecology
16S rRNA: 16 subunit of ribosomal ribonucleic acid
WPS‐2: Eremiobacteraeota

## 1 INTRODUCTION

Understanding of the gastrointestinal microbiota and its relevance to host health has provided new insight into gastrointestinal disease in animals. Advances in deoxyribonucleic acid (DNA) sequencing technology have enabled identification of bacterial species previously unrecognized using culture‐based techniques. Collectively known as the microbiota, the bacteria that reside in the gastrointestinal tract play a functional role in nutrient digestion and absorption, production of short chain fatty acids and conversion of bile acids, biosynthesis of vitamins and amino acids, regulation of the inflammatory environment of the gastrointestinal tract and immune modulation against pathogens.^1^

Changes in the bacterial community of the gastrointestinal tract that occur in association with disease states, termed dysbiosis, affect these physiologically important metabolic processes. In humans, dysbiosis has been associated with inflammatory bowel disease, ulcerative colitis, Crohn's disease, obesity, and metabolic disease.^2^ In companion animals such as dogs and cats, dysbiosis has been associated with acute and chronic enteropathies.^3^, ^4^, ^5^

Researchers studying horses have utilized similar molecular techniques in hopes of decreasing the substantial morbidity and mortality attributed to gastrointestinal disease in the horse.^6^ To date, studies have indicated that feces represent an adequate proxy for the hindgut of the horse,^7^ with 17 to 19 phyla identified and present in variable relative abundance.^8^, ^9^, ^10^, ^11^ Inherent factors such breed,^8^, ^11^, ^12^ age,^13^ and pregnancy status^14^ and external factors such as geographic location,^8^ transport,^15^ exercise intensity,^16^, ^17^, ^18^ fasting,^19^, ^20^ and season^21^ appear to have some influence on the fecal microbiome. Dietary variables such as exposure to pasture,^8^, ^22^ abrupt feed change,^22^ and feeding concentrate vs forage^23^ alter the microbiome to some extent, whereas the feeding of high starch concentrates^13^, ^24^ can induce gastrointestinal disease. To date, the factors with the greatest impact on the fecal microbiome include diet, antibiotic use,^25^, ^26^ and the presence of gastrointestinal disease such as colic or colitis.^27^, ^28^ Initial efforts to characterize the microbiome have been incomplete because of small sample size and use of university herds or horses at single locations, which may not adequately represent the equine population.^29^, ^30^, ^31^ Furthermore, because these factors have been described independently across studies, the size effects of these individual factors remain unclear as does whether the dietary effect is similar to that of disease of antibiotics.

Our purpose was to define the fecal microbiota in a large population of healthy horses to assess the influence of age, breed, sex, geographic location, season, dietary factors, and health status. We hypothesized that these individual or external factors would have relatively minor impact on the fecal microbiome when compared to alterations induced by gastrointestinal disease. We chose 2 common variants of colitis seen in our hospital population, horses with primary colitis attributable to *Salmonella* and those with colitis induced by antimicrobial use.

## 2 MATERIALS AND METHODS

No official animal care and use protocol was sought from the Texas A&M University Institutional Animal Care and Use Committee because all fecal samples were collected after natural elimination and clinical data were gathered historically.

### 2.1 Study population and sample collection

The study consisted of 2 populations of horses, healthy and those with colitis. All fecal samples were collected during the period of October 2015 to October 2018.

Healthy horses were sampled from nonhospital environments across multiple geographical locations in the United States. Fifteen ambulatory veterinarians were contacted by 1 investigator (C. E. Arnold) and asked to collect feces from healthy horses during routine wellness examinations. Veterinarians were instructed to sample no more than 2 horses per farm and collect feces from a minimum of 10 horses. The inclusion criteria for healthy horses consisted of the following: ≥1 year of age, no antibiotic or nonsteroidal anti‐inflammatory (NSAID) administration within 6 months, no history of colic or diarrhea within 6 months, and normal physical examination findings on the day of sample collection. Fecal samples were collected after natural defecation and stored at 4°C on the veterinary truck for ≤12 hours. Upon arrival at the clinic, samples were transferred to a −17°C freezer until shipping (typically 1 week). Samples were shipped frozen overnight to the Gastrointestinal Laboratory at Texas A&M University and kept frozen at −80°C until DNA extraction. Approximately 200 fecal samples from healthy horses were collected, and 80 samples were selected for sequencing. For the analysis of healthy horses, 80 samples were selected so as to create equal group sizes for diets A‐E, while maintaining representation from different breeds, ages, sexes, and geographic locations.

Fecal samples were collected from clinical patients with colitis admitted to the Veterinary Medical Teaching Hospital at Texas A&M in an effort to bank fecal samples from horses with gastrointestinal disease. Horses with antimicrobial‐associated diarrhea (AAD) were defined as those that had received antimicrobial prophylaxis before elective surgery or to treat a suspected or known infection before the development of diarrhea. Horses in the AAD group had no history of gastrointestinal disease before antibiotic administration, and the clinician of record classified colitis as being associated with antibiotic use. Horses in the AAD group received the following antimicrobial agents: ceftiofur crystalline (*n* = 4); metronidazole (n = 1); doxycycline (*n* = 1); penicillin and gentamicin (*n* = 3); penicillin, gentamicin, and doxycycline (*n* = 2); penicillin, gentamicin, and metronidazole (*n* = 2); and chloramphenicol (*n* = 1). Antimicrobials were used to treat known infections in 11 horses (respiratory, *n* = 6; lacerations, *n* = 2; cellulitis, *n* = 1; navicular bursoscopy, *n* = 1; sequestrum, *n* = 1) and as surgical prophylaxis in 3 horses (check ligament desmotomy, fasciotomy and neurectomy for proximal suspensory disease, and resection of a skin tumor under general anesthesia). Information regarding antimicrobial treatment of AAD horses is included in Supplementary Table 1.

Horses with *Salmonella* colitis were defined as those admitted to the Veterinary Medical Teaching Hospital at Texas A&M with a presenting complaint of colitis with no history of antimicrobial administration or prior gastrointestinal disease such as colic, positive polymerase chain reaction (PCR) test for *Salmonella* ^32^ and classified by the clinician of record as having *Salmonella* colitis.

Fecal samples from horses with colitis were defined as the first natural defecation upon admission and stored at 4°C until transported to the Gastrointestinal Laboratory at Texas A&M University for processing. Samples were stored at 4°C for ≤12 hours, and kept frozen at −80°C until DNA extraction.

The following information was collected for all horses: age, breed, sex, weight (estimated by the veterinarian), season of fecal collection, geographic location, and diet. Diet was analyzed by individual factors such as hay type, pasture type (warm season grasses such as Bermuda grass, Bahia or Buffalo pasture; transition zone grasses such as fescues or zoysia; cool season grasses such as Kentucky bluegrass and ryegrass), time spent in pasture (none, some or continuous), percentage maximum crude fiber in concentrate (low; 5%‐8%, medium; 10%‐15%, high; 18%‐33%), and amount of concentrate (none, 0.5% and 1%‐2% of body weight in kilograms per day). Finally, a dietary scale was created that included for all aspects of diet. Horses were categorized as 1 of the following: A, forage only (hay, pasture or both); B, forage plus low fiber concentrate (5%‐7%) fed at ≤0.5% of body weight in kg/day; C, forage plus medium fiber concentrate (10%‐15%) fed at ≤0.5% of body weight in kg/day; D, forage plus high fiber concentrate (18%‐33%) fed at ≤0.5% of body weight in kg/day; and, E, forage plus medium fiber concentrate (10%‐15%) fed at 1%‐2% of body weight in kg/day.

### 2.2 DNA extraction

One hundred milligrams of feces from the center of each fecal ball or liquid fecal sample were aliquoted into a sterile 1.7 mL tube (Microtube, Sarstedt AG & Co, Numbrecht, Germany) containing 150 μL of 0.1 mm zirconia‐silica beads and 100 μL of 0.5 mm zirconia‐silica beads (BioSpec Products Inc, Barlesville, OK, USA). Samples were homogenized (FastPrep‐24, MP Biomedicals, Irvine, CA, USA) for 1 minute at a speed of 4 m/s. The DNA was extracted using the PowerSoil DNA Isolation Kit (MO BIO, Carlsbad, CA, USA) following the manufacturer's instructions.

### 2.3 Sequencing of 16S rRNA genes

Sequencing of the V4 region of the 16S rRNA gene was performed at MR DNA (www.mrdnalab.com, Shallowater, TX, USA) using an Illumina MiSeq platform (Illumina Inc, San Diego, CA, USA). Following the manufacturer's guidelines, 2 × 300 paired‐end reads were produced using 515F (5′‐GTG YCA GCM GCC GCG GTA A‐3′) and 806R (5′‐GGA CTA CNV GGG TWT CTA AT‐3′) primers.^33^, ^34^ The PCR reaction was performed in a single‐step 30‐cycle PCR using the HotStarTaq Plus Master Mix Kit (Qiagen, Germantown, MD, USA) under the following conditions: 94°C for 3 minutes, followed by 28 cycles (5 cycles used on PCR products) of 94°C for 30 seconds, 53°C for 40 seconds and 72°C for 1 minute, after which a final elongation step at 72°C for 5 minutes was performed. Using Illumina TruSeq DNA's protocol, a DNA library was set up and Illumina MiSeq was used for sequencing according the manufacturer's guidelines.

### 2.4 Analysis of sequences

A total of 106 samples were analyzed, which generated 4 386 598 quality sequences. Sequences were analyzed using a QIIME 2 (Quantitative Insights into Microbial Ecology)^35^ v.2019.7 pipeline as described elsewhere.^36^, ^37^ Briefly, barcodes and primers were removed and short (<150 bp), ambiguous, homopolymeric sequences were depleted from the dataset. The program divisive amplicon denoising algorithm (DADA2) was used to identify and remove chimeric sequences.^38^ The amplicon sequence variant (ASV) table was created using DADA2,^39^ and rarefied to 41 383 sequences per sample based on the lowest read depth in all samples for even depth of analysis. Sequences determined to be mitochondria, chloroplasts, unassigned, or those belonging to the phylum cyanobacteria were excluded from further analysis. Data were deposited in the National Center for Biotechnology Information Sequence Read Archive under the accession number SRP228480 and BioProject number PRJNA580257.

Alpha diversity metrics Chao1 (richness), observed ASVs (species richness), and Shannon diversity (evenness) were generated in QIIME2. Beta diversity was evaluated with the phylogeny‐based weighted and unweighted UniFrac distance metric, and Principal Coordinate Analysis (PCoA) plots for visualization were generated in QIIME2.

### 2.5 Statistical analysis

Before analysis, data was tested for normality using the Shapiro‐Wilk test (JMP Pro 14, SAS, Marlow, Buckinghamshire, UK). Because data were not normally distributed, nonparametric measures were used throughout the study.

Beta diversity (bacterial community composition) was measured using weighted and unweighted UniFrac metrics and visualized for clustering using Principle Coordinate Analysis (PCoA) plots. An analysis of similarity test (ANOSIM) within the PRIMER 6 (PRIMER‐E Ltd, Luton, UK) software package was performed on the distance matrices to assess the significance of the differences in the bacterial community composition‐based individual factors (age, breed, sex, geographic location, season, dietary variables, and categories A‐E) for healthy horses and later for a comparison of healthy horses to those with colitis. The resulting *R* values were defined as follows: *R* < 0.1 similar; *R* = 0‐0.25, similar with high overlap; *R* = 0.25‐0.5, different with some overlap; *R* = 0.5‐0.75, different; *R* = 0.75‐1, highly different.

Statistical analysis of alpha diversity indices (Chao 1, observed ASVs, and Shannon) was performed using the software package PRISM (PRISM 8, GraphPad Software Inc, San Diego, CA, USA). A Kruskal‐Wallis test with Dunn's multiple comparison test was used to examine the effects of individual factors (age range [1‐5, 6‐10, 11‐15, 16‐20, 21‐25 years old], sex, season, and dietary variables [percentage maximum crude fiber in concentrate, amount of concentrate, time in pasture, hay type, pasture type, and diet classification scale]) on alpha diversity measures in healthy horses. A 1‐way analysis of variance and Tukey's post‐test were used to analyze breed and geographic location. A Kruskal‐Wallis test with a Dunn's multiple comparison test were used later to compare the alpha diversity metrics of healthy horses to those with colitis.

Univariate analysis of the relative abundance of the bacterial taxa in the fecal samples was evaluated using a Kruskal‐Wallis test (PRISM 7, GraphPad Software Inc, San Diego, CA, USA) followed by a Dunn's multiple comparison post‐test. Only bacterial taxa present in at least 50% of the samples were included in the analysis.

Linear discriminant analysis effect size (LEfSe) using the web‐based program Calypso v8.62 (http://cgenome.net/wiki/index.php/Calypso) was performed to analyze the relative abundance of bacterial taxa and their associations with any of the 5 diet categories. A cutoff threshold of 3.5 was set for significance.

## 3 RESULTS

### 3.1 Study participants

Breed, age, sex, and diet of the healthy horses and those with colitis are summarized in Supplementary Figures 1‐4. Supplemental Table 1A contains information regarding signalment, diet, season of collection, geographic location, individual dietary components, and estimated weight of all healthy horses.

### 3.2 The effect of diet A‐E on the fecal microbiome of healthy horses

#### 3.2.1 Beta‐diversity measures

**FIGURE 1:**
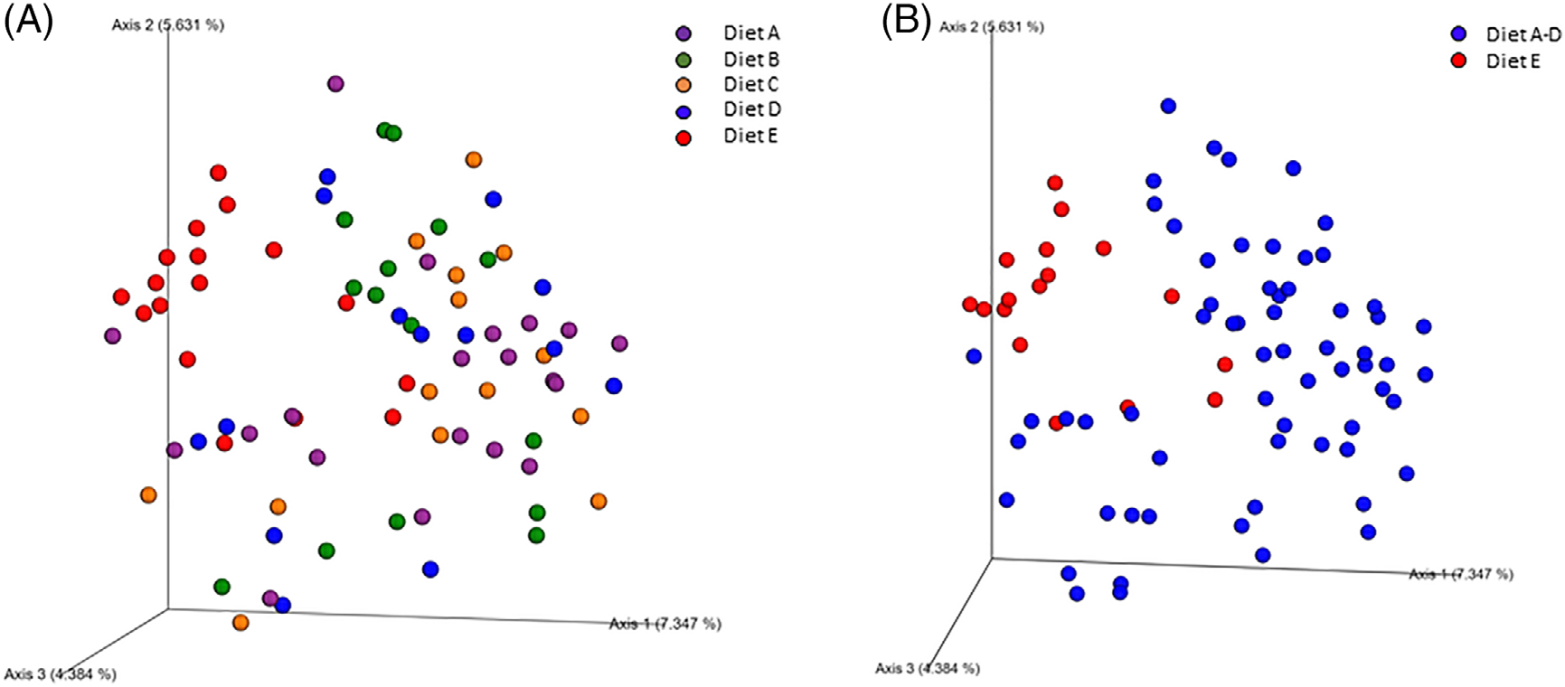
Principal coordinates analysis (PCoA) plots based on unweighted UniFrac distances showing mild differences between healthy horses on diet E compared to diets A‐D (ANOSIM, *R* = 0.156, *P* = .05) of healthy horses by diet. A, Horses fed forage only (hay and/or pasture), purple; B, horses fed forage plus low fiber concentrate (5%‐7%) at ≤0.5% of body weight in kg/day, green; C, forage plus medium fiber concentrate (10%‐15%) fed at ≤0.5% of body weight in kg/day, orange; D, forage plus high fiber concentrate (18%‐33%) fed at ≤0.5% of body weight in kg/day blue; E, forage plus medium fiber concentrate (10%‐15%) fed at 1% to 2% of body weight in kg/day, red. B, Horses on diets A‐D combined (blue) show mild clustering from those on diet E (red) (ANOSIM, *R* = 0.15, *P* = .03)

A principal coordinate analysis plot of unweighted UniFrac distances in normal horses by diet category is shown in Figure 1. Figure 1A indicates some overlap among horses on each diet (ANOSIM, unweighted, *R* = 0.156, *P* = .05) whereas Figure 1B indicates some distinction between horses on diet E and those combined on diets A‐D (ANOSIM, *R* = 0.15, *P* = .03). Supplemental Table 2 contains the pairwise comparison of the unweighted and weighted UniFrac distances by diet category.

#### 3.2.2 Alpha diversity measures

The alpha diversity metrics of healthy horses as stratified by diet category are presented in Supplementary Figure 5. Neither ASVs, Chao 1, nor Shannon metrics showed statistical significance among diets A‐E.

#### 3.2.3 Taxonomy

**FIGURE 2:**
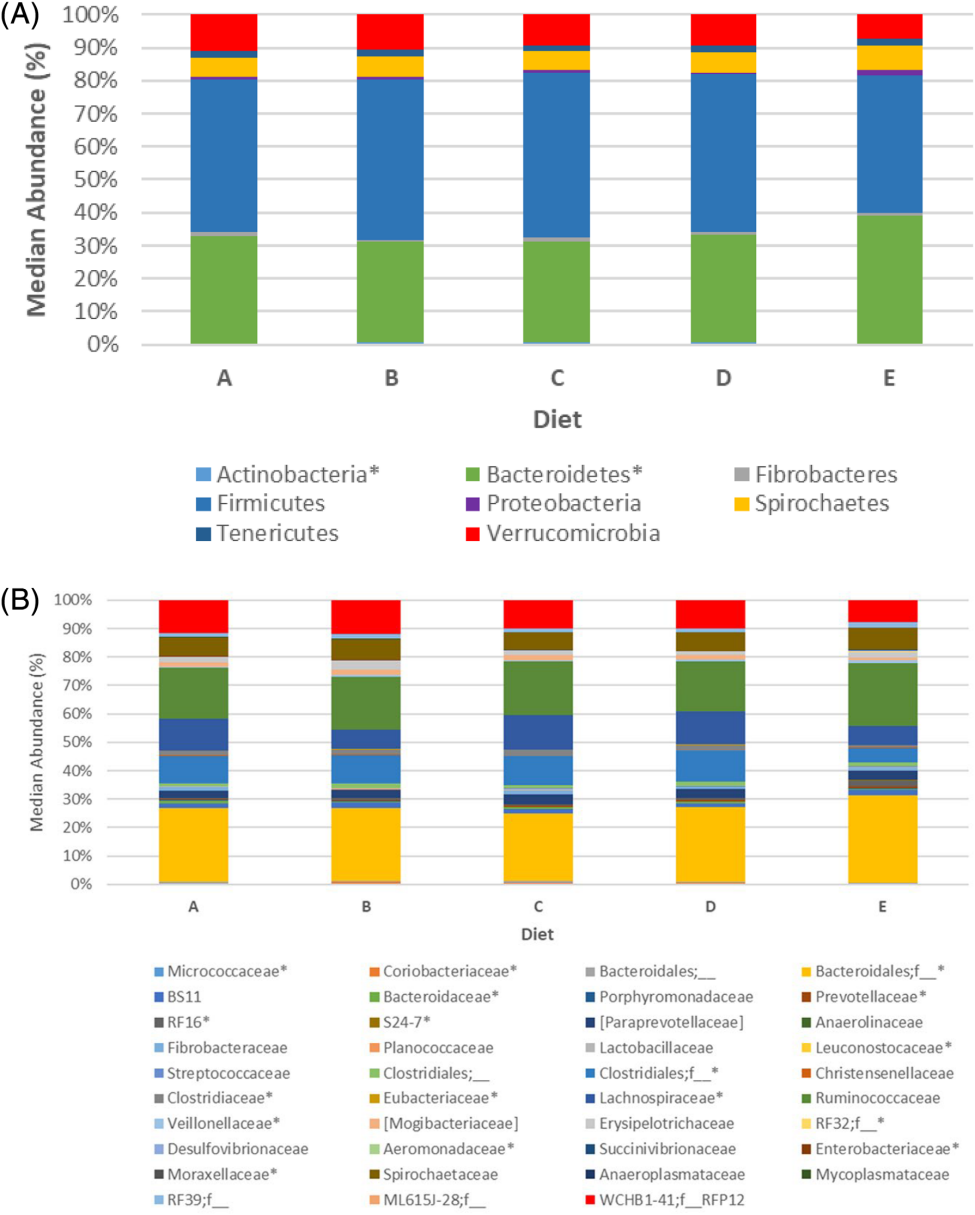
Microbial composition of healthy horses fed diets A‐E at the (A) phylum and (B) family level. * denotes taxa with significant differences between groups (*P* < .05)

Information regarding the taxa of horses stratified by diet category at the phylum, family, and species levels can be found in Supplementary Tables 3 to 5. Histograms depicting the median abundance of each phylum for horses on diets A‐E can be found in Figure 2. At the phylum level, 4 taxa were significantly altered by diet, but only Actinobacteria remained statistically significant after adjustments for multiple comparisons were made. Actinobacteria were decreased in the diet E compared to diets B‐D (*P* = .003, *Q* = 0.04). At the family level, only *Micrococcaceae* from the phylum Actinobacteria, class Actinobacteria was decreased in diet E (*P* = .01, *Q* = 0.03) whereas the class Coriobacteriia, family *Coriobacteriaceae* was not significant after adjustments for multiple comparisons were made (*P* = .04, *Q* = 0.10).

**FIGURE 3:**
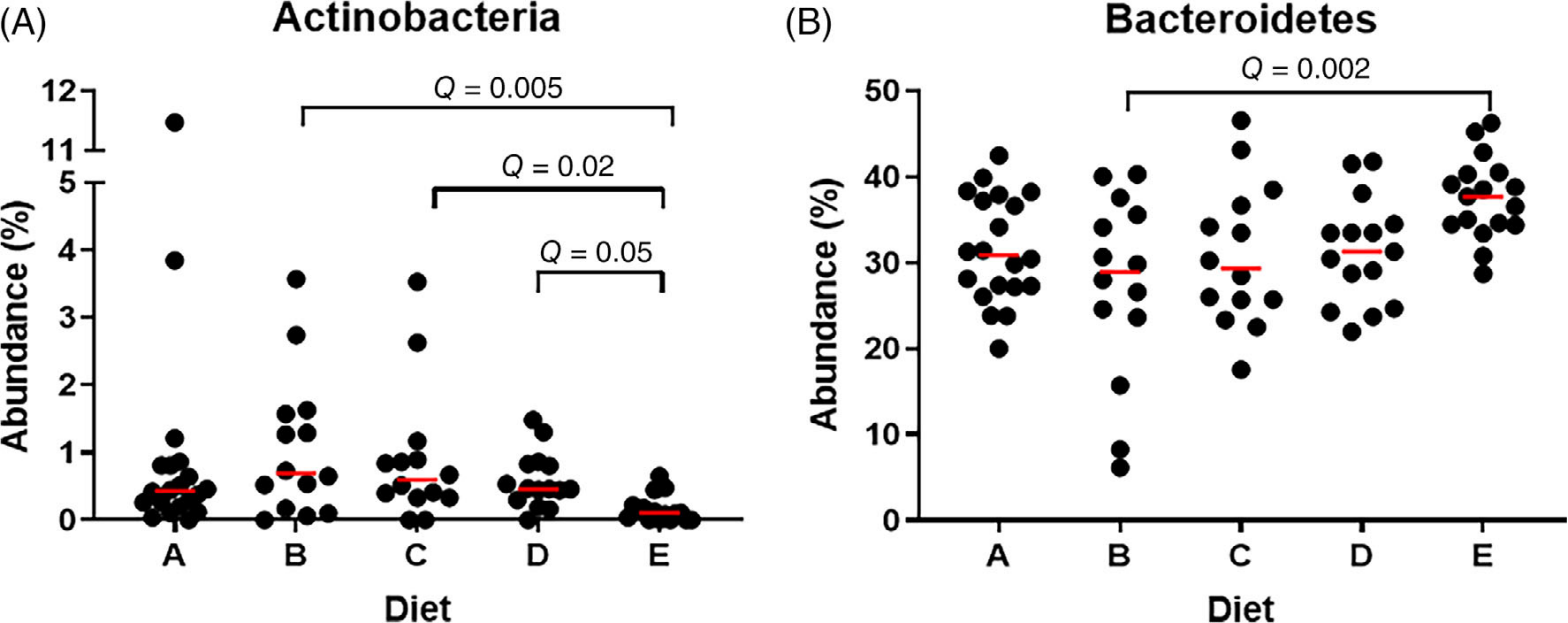
The median abundance (%) of significantly altered phyla according to linear discriminant effects size analysis of healthy horses on diets A‐E. A, Horses on diet E have significantly less Actinobacteria than those on diets B, C, and D. B, Horses on diet E have significantly more Bacteroidetes than those on diet B

Linear discriminant effects size analysis (LEfSe) analysis at the phylum level indicated that Actinobacteria were associated with diet A whereas Bacteroidetes and Eremiobacteraeota (WPS‐2) were associated with diet E. Figure 3 indicates the median abundance of Actinobacteria and Bacteroidetes in each horse in the respective dietary groups. Eremiobacteraeota is not included because of its low prevalence in the population despite significance on LEfSe analysis. Supplementary Figure 6 contains the results of LEfSe analysis at the phylum and family level.

### 3.3 The effect of signalment and external factors on the fecal microbiome of healthy horses

#### 3.3.1 Beta‐diversity measures

The following variables achieved significance after ANOSIM testing: breed (unweighted, *R* = 0.19, *P* = .0001), state (unweighted, *R* = 0.29, *P* = .001; weighted, *R* = 0.17, *P* = .002), season (unweighted, *R* = 0.02, *P* = .002; weighted, *R* = 0.10, *P* = .04), percentage maximum crude fiber in the concentrate (unweighted, *R* = 0.05, *P* = .05), amount of concentrate (unweighted, *R* = 0.287, *P* = .002; weighted, *R* = 0.132, *P* = .05), and time in pasture (unweighted *R* = 0.06, *P* = .02). Results of ANOSIM analysis of unweighted and weighted UniFrac distances for all individual variables are presented in Table 1.

**TABLE 1:** The results of ANOSIM testing for individual variables in healthy horses using weighted and unweighted UniFrac distances

	Unweighted		Weighted	
	*R* statistic	*P* value	*R* statistic	*P* value
Age range	0.006	.43	0.029	.25
Breed	0.191	<.001	0.162	.08
Sex	0.007	.4	0.004	.39
State	0.29	.001	0.172	.002
Season	0.017	.002	0.1	.04
% fiber	0.058	.05	0.047	.09
Amount concentrate	0.287	.002	0.132	.05
Time in pasture	0.061	.02	0.023	.19
Pasture zone	0.042	.1	0.038	.13
Hay type	0.008	.43	−0.08	.54

#### 3.3.2 Alpha‐diversity measures

Of all the variables analyzed, only sex and time in pasture had any significant effect on alpha diversity measures (Supplemental Figures 7 and 8). Mares had lower diversity than geldings but not stallions for the Chao 1 (*P* = .05) and observed ASVs (*P* = .05) metrics, but significance was lost after correction for multiple comparisons (*Q* = 0.07) for both. Lack of pasture exposure had the lowest of all 3 diversity indices compared to some pasture (Chao 1, *P* = .01, *Q* = 0.03; observed ASVs, *P* = .01, *Q* = .03; Shannon, *P* = .02, *Q* = 0.03). The effect of other individual diet‐related variables was not significant. Alpha diversity metrics for all individual values are listed in Supplementary Table 6.

### 3.4 The effect of colitis associated with AAD and *Salmonella* on the fecal microbiome

A population of clinical patients with colitis associated with AAD (*n* = 14) and *Salmonella* (*n* = 12; Supplementary Table 1B) was compared to healthy horses previously described.

#### 3.4.1 Diversity between samples (beta diversity)

**FIGURE 4:**
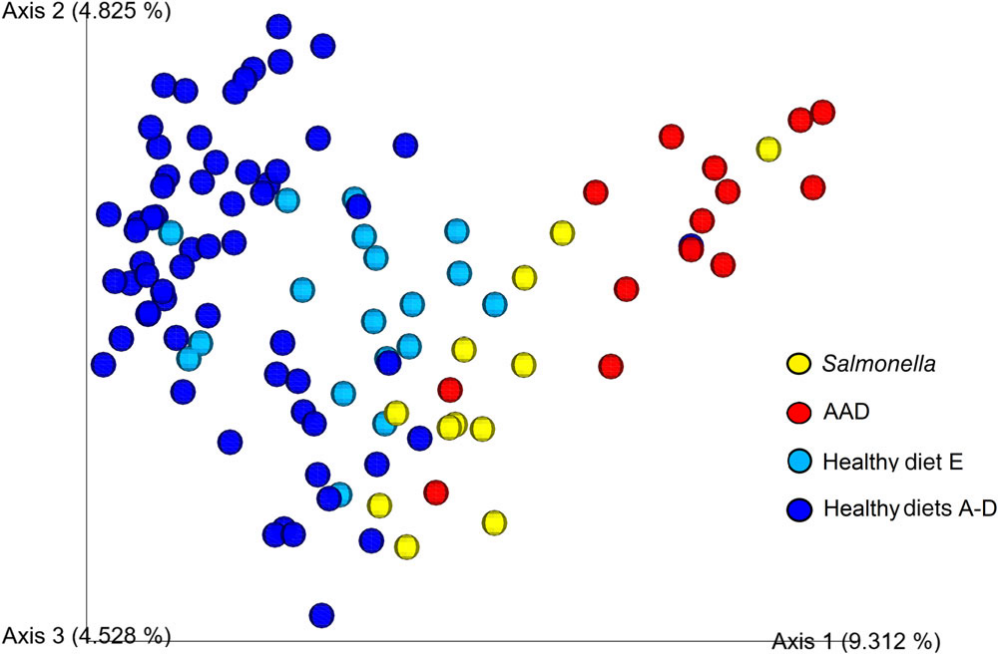
Principal coordinate analysis plot of unweighted UniFrac distances shows distinct clustering of horses with antimicrobial‐associated diarrhea (AAD) (red) and *Salmonella* (yellow) colitis from healthy horses on diets A‐D (royal blue) and diet E (light blue) (overall ANOSIM, *R* = 0.565, *P* < .001). There is statistically significant but less distinct separation of the 2 types of colitis horses from each other (AAD vs *Salmonella*; ANOSIM, *R* = 0.226, *P* < .001) and healthy horses on diets A‐D from those on diet E (ANOSIM, *R* = 0.287, *P* < .001)

A principal coordinate analysis plot of unweighted UniFrac distances is presented in Figure 4, and indicates significant clustering between healthy horses and those with colitis (overall ANOSIM, *R* = 0.565, *P* = .0001). Pairwise tests (Table 2) indicates that AAD horses were significantly separated from healthy horses on diets A‐D (ANOSIM, *R* = 0.861, *P* = .0001) and diet E (ANOSIM, *R* = 0.679, *P* = .0001). Healthy horses on diets A‐D (ANOSIM, *R* = 0.608, *P* = .0001) and diet E (ANOSIM, *R* = 0.581, *P* = .0001) had significantly different microbial community composition from horses with *Salmonella*. Less separation was found between horses with AAD and *Salmonella* colitis (*R* = 0.0226, *P* = .009). Results of ANOSIM testing for weighted UniFrac values are listed in Table 2.

**TABLE 2:** Pairwise ANOSIM analysis of unweighted and weighted UniFrac distances in healthy horses (diets A‐D and diet E) and those with antimicrobial‐associated diarrhea (AAD) and *Salmonella* colitis

Groups	Unweighted		Weighted	
	*R* statistic	*P* value	*R* statistic	*P* value
Overall	0.565	<.001	0.488	<.001
Healthy A‐D, AAD	0.861	<.001	0.781	<.001
Healthy E, AAD	0.679	<.001	0.503	<.001
Healthy A‐D, *Salmonella*	0.608	<.001	0.467	<.001
Healthy E. *Salmonella*	0.581	<.001	0.363	.001
AAD, *Salmonella*	0.226	<.001	0.201	<.001
Healthy A‐D, healthy E	0.287	<.001	0.135	.05

#### 3.4.2 Diversity within samples (alpha diversity)

**FIGURE 5:**
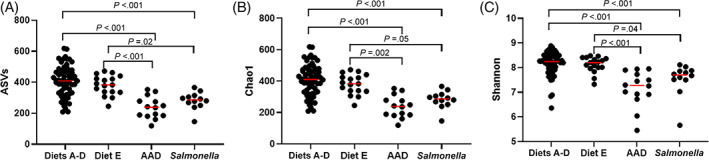
Alpha diversity measures of healthy horses with antimicrobial‐associated diarrhea (AAD) and *Salmonella* colitis. Amplicon sequence variants (ASVs) (A), Chao1 (B), and Shannon (C) indices are significantly reduced in both colitis groups (AAD and *Salmonella*) compared to both dietary groups of healthy horses, but not to each other

Alpha diversity indices were significantly lower in horses with colitis compared to healthy horses (Figure 5), but horses with AAD and *Salmonella* were not different from each other. Observed ASVs were decreased in healthy horses compared to horses with AAD (diets A‐D, *P* < .0001; diet E, *P* = .001) and *Salmonella* (diets A‐D, *P* = .001; diet E, *P* = .02), but no difference was found between horses with each type of colitis. Chao 1 was significantly different between healthy horses and those with AAD (diets A‐D, *P* < .0001; diet E, *P* = .02) and *Salmonella* (diets A‐D, *P* = .0004; diet E, *P* = .05), whereas no difference was detected between the horses with either form of colitis. Finally, the Shannon metric was decreased between healthy horses and those AAD (diets A‐D, *P* < .0001; diet E, *P* = .009) and *Salmonella* (diets A‐D, *P* < .0009; diet E, *P* = .38) but not between the 2 colitis groups (*P* = .10).

#### 3.4.3 Taxonomy

**FIGURE 6:**
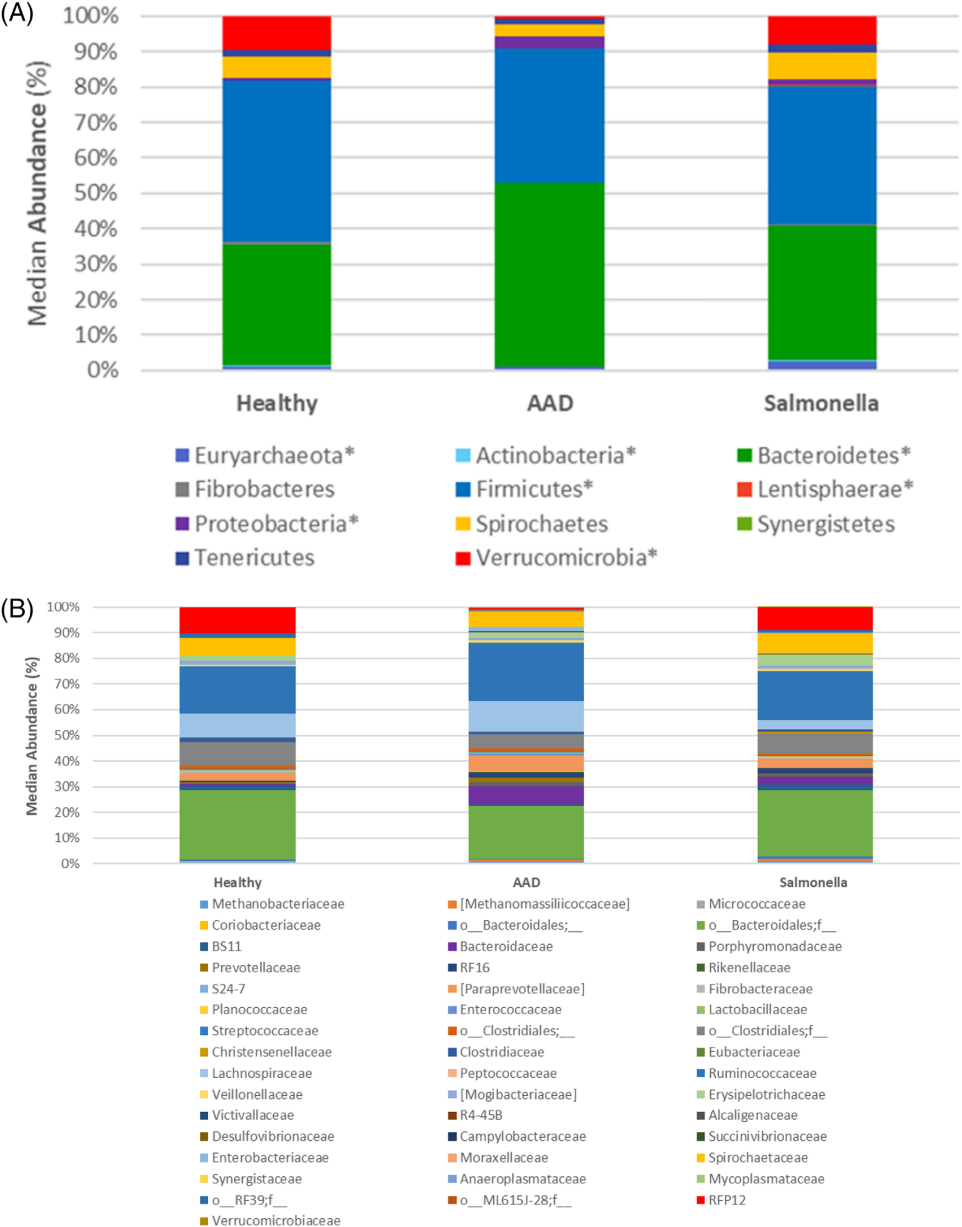
Microbial composition of healthy horses and those with colitis because of antimicrobial use (antimicrobial‐associated diarrhea [AAD]) and *Salmonella* at the levels of (A) phylum and (B) family. * denotes taxa with significant differences between groups (*P* < .05)

Supplementary Tables 7 to 9 contains the median bacterial abundances and ranges for healthy horses and those with AAD and *Salmonella* colitis at the phylum, family, and species levels. A histogram displaying the median bacterial abundance at the phylum and family levels can be found in Figure 6. At the phylum level, 7 phyla that had significant changes between healthy horses and those with colitis.

Horses with *Salmonella* had increased Euryarchaeota compared to healthy horses (*P* = .002, *Q* = 0.02) but not compared to horses with AAD. The family *Methanomassiliiococcaceae* was increased in the AAD and *Salmonella* groups (*P* = .0001, *Q* = 0.0007) compared to healthy horses.

Actinobacteria were not significantly different across groups (*P* = .03, *Q* = 0.05), but the family *Micrococcaceae* was increased in healthy horses (*P* = .005, *Q* = 0.0130).

Horses with AAD had significantly more Bacteroidetes (*P* = .0004, *Q* = 0.0015) compared to healthy horses but not those with *Salmonella*. Within the phylum Bacteroidetes, the majority of this change occurred within the order Bacteroidales and an unassigned family (*P* = .006, *Q* = 0.0136) that was decreased in the AAD horses compared to healthy horses and those with *Salmonella*. Other more minor changes at the family level involved an increase in *Bacteroidaceae* (*P* = .0001, *Q* = 0.0007), *Rikenellaceae* (*P* = .0001, *Q* = 0.0007), and *Porphyromonadaceae* (*P* = .0001, *Q* = 0.0007) in the 2 colitis groups compared to healthy horses. In contrast, the family BS11 was decreased in the AAD group compared to healthy horses (*P* = .0001, *Q* = 0.0007).

Horses with *Salmonella* had significantly less Firmicutes than horses in the healthy groups (*P* = .008, *Q* = 0.0176). Within the class Bacilli, the family *Enterococcaceae* was increased in AAD horses (*P* = .0001, *Q* = 0.0007), whereas *Lactobacillaceae* were increased in the healthy group compared to horses with *Salmonella* (*P* = .05, *Q* = 0.07). Within the class Clostridia an unknown group within the order *Clostridiales* was decreased in the *Salmonella* group but not in the healthy or AAD groups (*P* = .01, *Q* = 0.01). An unknown family within the order *Clostridiales* (*P* = .002, *Q* = 0.01) and *Mogibacteriaceae* (*P* = .0002, *Q* = 0.001) were significantly decreased in the AAD group but not in the healthy or *Salmonella* groups. *Lachnospiraceae* were significantly decreased within the *Salmonella* group compared to healthy controls and AAD horses (*P* = .0006, *Q* = 0.003). *Eubacteriaceae* were increased in healthy horses compared to those with AAD (*P* = .0004, *Q* = 0.002).

Lentisphaerae were increased in the *Salmonella* group (*P* = .001, *Q* = 0.0036) compared to healthy horses and those with AAD. Two families within the class [Lentisphaeria], *Victivallaceae* (*P* = .04, *Q* = 0.06) and *R4‐45B* (*P* = .003, *Q* = 0.009) accounted for this increase.

Overall, Proteobacteria were increased in the AAD group compared to the *Salmonella* and healthy groups (*P* = .0002, *Q* = 0.001). Within the class Betaproteobacteria, the family *Alcaligenaceae* was increased in the AAD and *Salmonella* groups compared to healthy horses (*P* = .001, *Q* = 0.005), whereas *Campylobacteriaceae* from the class Epsilonproteobacteria were increased only in the *Salmonella* groups (*P* = .01, *Q* = 0.03). Within the class Gammaproteobacteria, *Enterobacteriaceae* were increased in AAD horses (*P* = .0001, *Q* = 0.0007) compared to healthy horses and *Moraxellaceae* (*P* = .01, *Q* = 0.03) were decreased in AAD horses compared to healthy horses.

The phylum Verrucomicrobia was significantly decreased in the AAD group compared to healthy horses and those with *Salmonella* (*P* = .0001, *Q* = 0.001). This change is a result of a marked depletion of RFP12 (*P* = .0001, *Q* = 0.0007) from the class Verrucomicrobiae in horses with AAD compared to healthy horses and those with *Salmonella*, whereas a small increase was found in the family *Verrucomicrobiaceae* within the class Verrucomicrobiae (*P* = .005, *Q* = 0.01) in horses with both types of colitis compared to healthy horses.

**FIGURE 7:**
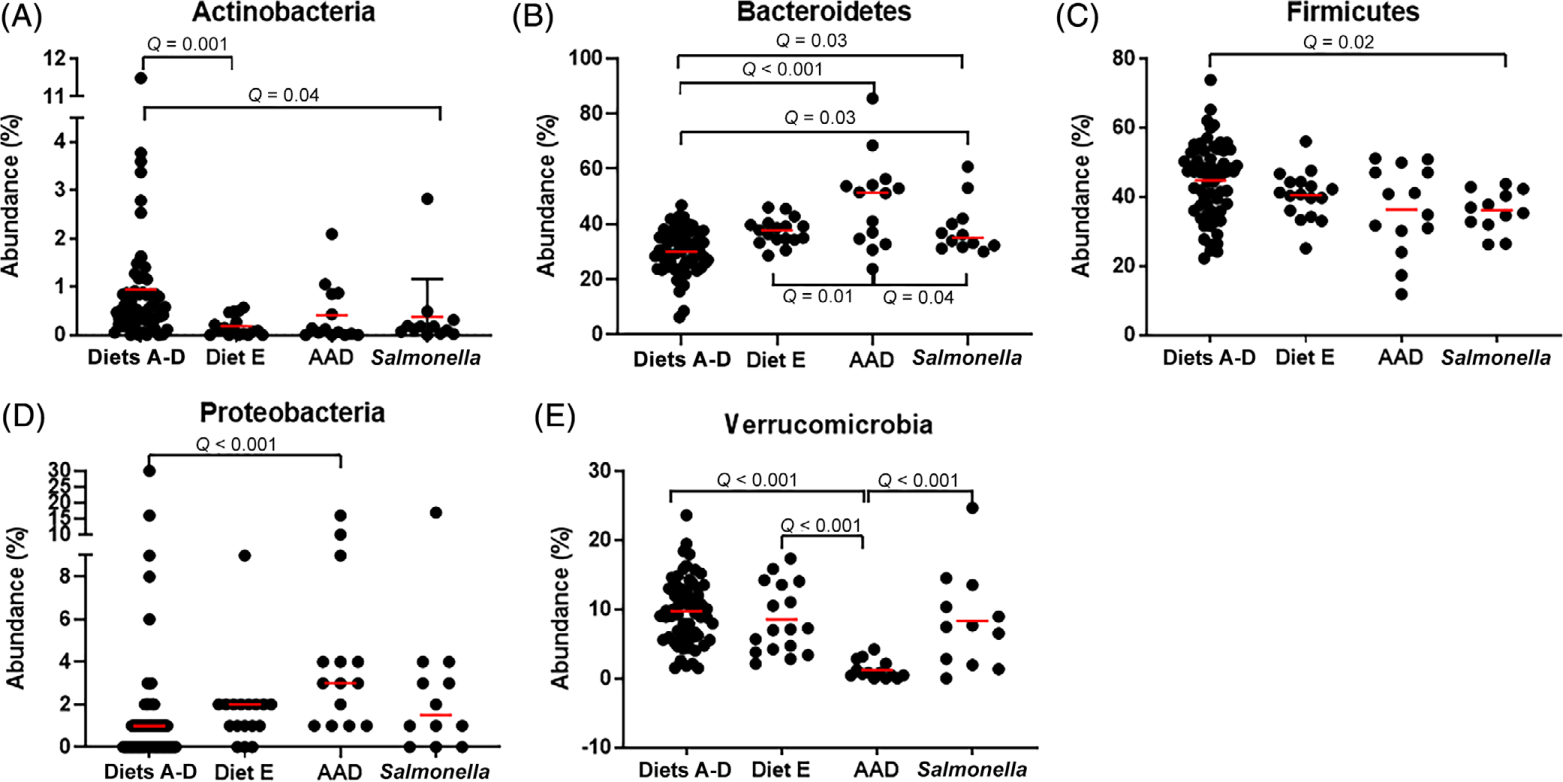
The median abundance (%) of significantly altered phyla according to linear discriminant effects size analysis in healthy horses and those with antimicrobial‐associated diarrhea (AAD) and *Salmonella*. Healthy horses are separated by diets A‐D and diet E. A, The abundance of Actinobacteria is significantly greater in healthy horses on diets A‐D than those on diet E and for those with *Salmonella* colitis. B, Horse with AAD have significantly more Bacteroidetes than healthy horses on diets A‐D or diet E and those *Salmonella*. C, Horses with *Salmonella* colitis have significantly less Firmicutes than healthy horses on diets A‐D. D, Horses with AAD have significantly more Proteobacteria than healthy horses on diets A‐D. E, Horses with AAD have a marked reduction in the amount of Verrucomicrobia compared to healthy horses on diets A‐D and diet E and *Salmonella* colitis

The abundance of phyla found significantly different among the 5 groups on LEfSe analysis is presented in Figure 7. Healthy horses on diets A‐D had more Actinobacteria than did those on diet E and those with *Salmonella* colitis. Horses with AAD had more Bacteroidetes and Proteobacteria, whereas horses with *Salmonella* had more Fusobacteria, Euryarchaeota, and Lentisphaerae. Healthy horses had more Firmicutes, Verrucomicrobia, WPS‐2 and Absconditabacteria (SR1). The results of LEfSe analysis is found in Supplementary Figure 9.

### 3.5 Enteric pathogens

Based on the inclusion criteria, none of the horses in the AAD or *Salmonella* groups had a previous history of gastrointestinal disease such as colic. The 12 horses in the *Salmonella* group were positive for *Salmonella* on quantitative PCR (qPCR). Of the 14 horses in the AAD group, 9 were positive for *Salmonella* on qPCR. For the remaining 5 horses, *Salmonella* testing was not requested by the attending clinician. The results of enteric pathogen testing performed for each horse are presented in Supplemental Table 1.

## 4 DISCUSSION

Our study characterized the fecal microbiome of healthy adult horses by sampling from a large, diverse equine population not associated with single farms or university teaching herds. A stringent set of inclusion criteria ensured participation of healthy individuals. A veterinarian familiar with the horse was asked to perform a physical examination, estimate weight, confirm dietary information, and exclude horses with recent history of NSAID or antibiotic use, and gastrointestinal disease.

Not all aspects of the study were strictly controlled. With regard to diet, a proper feed analysis was not performed on each patient because of practicality and cost. Veterinarians estimated the weights of healthy horses, which may have introduced error. Although quantity and fiber percentage were used to stratify concentrates, no attempt was made to assess the quantity or content of forages or the use of dietary supplements. Diet and exercise were confounded for horses in diet E, elite level athletes that ate 2 to 4 times the concentrate compared to horses on diets A‐D. Finally, some horses in the AAD group underwent general anesthesia or standing sedation during surgery and were administered NSAIDs. All of these factors can affect microbiome composition^16^, ^17^, ^18^, ^19^, ^40^ and should be considered when interpreting our results.

Statistical analysis of the healthy horse population indicated that inherent factors such as age, breed, and sex had a minor impact on the fecal microbiome compared to colitis. Unlike previous studies, advanced age^13^, ^41^ did not significantly affect the fecal microbiome of the horses in our study. Breed showed a mild but statistically significant clustering on PCoA plot with no significant effect noted on alpha diversity metrics. Sex had an impact on richness indices (mares had decreased alpha diversity metrics compared to geldings), but no effect was seen on beta diversity. Similar results have been reported in the human medical literature with females having less richness than males.^42^ Because geldings have very low concentrations of testosterone because of castration, this effect may be a result of the influence of estrogen and progesterone. Because of the limited impact of these variables on diversity measures, their effect on taxonomy was not investigated.

Environmental factors that were studied included state of residence, season, and diet. Healthy horses displayed mild but significant clustering by state on PCoA plot, although this factor is confounded by other variables such as type of hay or pasture, athletic occupation, and differences of sample size among states. There was a significant lack of clustering by season of sample collection on PCoA plot. Samples from healthy horses were collected only during the winter, spring, and summer months. We speculate that this lack of effect in 3 seasons also would apply to the fall. Diet was examined by individual variables that included the type and amount of forages and concentrate before the development of the inclusive dietary scale. Three dietary factors showed significance on ANOSIM testing: amount of concentrate, maximum percentage of fiber in the concentrate, and time spent in pasture. Only the amount of concentrate showed significant clustering on PCoA plots with horses fed high amounts of grain (1%‐2% of body weight in kg/day) showing separation from those horses fed either no grain or <0.5% of body weight in kg/day. The variable time spent in pasture had significant effects on both alpha (horses on some pasture had increased richness and evenness compared to those on no pasture) and beta diversity (no significant clustering), but because these effects were mixed they are difficult to interpret.

A dietary scale that accounted for the feeding of forages and concentrates was created to stratify horses based on common feeding practices. Horses on diet A ate only forages, whereas those on diets B‐D ate a combination of forages and amounts of concentrate suitable for horses in light to moderate athletic activity. These groups ate concentrate containing a low, moderate, and high percentage of maximum crude fiber. Horses fed diet E were elite athletes in intense exercise training, specifically, Thoroughbred racehorses. These horses were fed a medium fiber containing concentrate similar to that of group C, but at 2 to 4 times the amount. Stratification of healthy horses by dietary group resulted in mild differences in beta diversity and taxonomy between diets E and B‐D. Horses on diet E tended to cluster separately from the other groups on PCoA plot of unweighted UniFrac distances, and had decreased abundance of Actinobacteria, a finding which has been reported in horses fed high starch diets.^13^, ^43^ Horses fed diet E had increased amounts of Bacteroidetes on LEfSe analysis, an effect that did not reach significance on univariate analysis. These results suggest that horses fed 1% to 2% of body weight in kilogram of concentrate have differences in bacterial community composition.

The group of horses fed diet E comprised a much more homogenous population compared to those on diets A‐D. Horses in group E were young Thoroughbreds at training centers or racetracks in New York, Kentucky, and Florida, consuming 2 to 4 times the amount of concentrate fed to other horses. It is possible that differences in the fecal microbiome of horses on diet E were due not to diet alone, but to similarity in breed, age, or exercise intensity. To investigate if breed and age accounted for these differences, ANOSIM testing was performed separately for breed (Thoroughbred) and age group (1‐5 years) between horses on diets A‐D vs diet E. The lack of significant differences between these variables in the 2 groups indicates that fecal microbiome differences may be associated with diet or exercise intensity. Because previous studies have found the bacterial community composition changes are associated with the initiation of exercise and return to baseline upon adaptation,^16^, ^17^, ^18^ it is likely that diet alone is responsible for the changes in beta diversity and taxonomy of horses fed diet E.

We chose to compare the fecal microbiome of healthy horses to that of horses with AAD and *Salmonella*. These are common variants of colitis in our hospital population and their effects on the fecal microbiome are as yet undescribed in the literature. According to the case definition, diarrhea was defined as spontaneous in onset with a positive qPCR test for *Salmonella* or secondary to antibiotic use. We did not stratify the AAD group further with enteric pathogen testing results. Because of this case definition, 5 horses in the AAD group and all of the healthy horses were not tested for enteric pathogens. Future studies should consider characterizing AAD cases according to results of enteric pathogen testing.

The presence of gastrointestinal disease markedly affected the diversity and composition of the fecal microbiome. Horses with each type of colitis had marked decreases in each of the alpha diversity measures (observed ASVs, Chao 1, and Shannon) compared to the 2 groups of healthy horses. However, each subset of healthy horses and colitis horses was not significantly different from each other with regard to richness and evenness. Microbial community composition however indicated large differences between healthy horses and those with colitis, with smaller yet significant differences within the subsets of healthy (diets A‐D vs E) and colitis (AAD vs *Salmonella*) horses. This can be seen on PCoA plots with AAD and *Salmonella* horses clustering distinctly from healthy horses, and with the AAD group having greater distance (ie, a more different microbiome composition) from healthy horses than those with *Salmonella*.

Horses with colitis had significant changes in the bacterial community composition of the fecal microbiome with 7 major phyla affected (Euryarchaeota, Actinobacteria, Bacteroidetes, Firmicutes, Lentisphaerae, Proteobacteria, and Verrucomicrobia). The results of univariate analysis found distinct microbial patterns associated with each type of colitis. Horses with AAD had increases in Bacteroidetes and Proteobacteria and decreases in Verrucomicrobia compared to healthy horses. Horses with *Salmonella* had decreases in Firmicutes and increases in Euryarchaeota and Lentisphaerae compared to healthy horses. These results were supported by the LEfSe analysis. To date, the fecal microbiome of horses with colitis only has been described in a population of horses with undifferentiated colitis. Although these horses also had significant decreases in richness and evenness, they experienced changes in the percentages of Bacteroidetes and Firmicutes simultaneously. These results suggest that colitis associated with antimicrobial use or *Salmonella* may have different effects on the fecal microbiome composition. Additional investigation utilizing a population of healthy horses and those with colitis better characterized by enteric pathogen testing, and potentially stratified by antimicrobial agent for AAD horses, is warranted.

The presence of colitis caused by AAD and *Salmonella* produced marked dysbiosis of the fecal microbiome with different effects on major phyla. Although both colitis groups clustered apart from healthy horses, horses with AAD had a larger shift in microbiome composition than did horses with *Salmonella*, compared to healthy controls. The effect of gastrointestinal disease was larger than that produced by diet.

## CONFLICT OF INTEREST DECLARATION

Authors declare no conflict of interest.

## OFF‐LABEL ANTIMICROBIAL DECLARATION

Authors declare no off‐label use of antimicrobials.

## INSTITUTIONAL ANIMAL CARE AND USE COMMITTEE (IACUC) OR OTHER APPROVAL DECLARATION

Authors declare no IACUC or other approval was needed.

## HUMAN ETHICS APPROVAL DECLARATION

Authors declare human ethics approval was not needed for this study.

## Supporting information

**Table S1** Supporting information

**Figure S1** Supporting information
